# Reduction of CTRP9, a novel anti-platelet adipokine, contributes to abnormal platelet activity in diabetic animals

**DOI:** 10.1186/s12933-015-0321-1

**Published:** 2016-01-11

**Authors:** Wenqing Wang, Wayne Bond Lau, Yajing Wang, Xinliang Ma, Rong Li

**Affiliations:** Department of Hematology, Tangdu Hospital, The Fourth Military Medical University, 710038 Xian, People’s Republic of China; Department of Emergency Medicine, Thomas Jefferson University, 1025 Walnut Street, Philadelphia, PA 19107 USA; Department of Geriatrics, Xijing Hospital, The Fourth Military Medical University, 710032 Xian, People’s Republic of China

**Keywords:** Diabetes, Adipokine, Platelet

## Abstract

Platelet hyper-reactivity is a crucial cause of accelerated atherosclerosis increasing risk of thrombotic vascular events in diabetic patients. The mechanisms leading to abnormal platelet activity during diabetes are complex and not fully defined. The current study attempted to clarify the role of CTRP9, a novel adiponectin paralog, in enhanced platelet activity and determined whether CTRP9 may inhibit platelet activity. Adult male C57BL/6 J mice were randomized to receive high-fat diet (HFD) or normal diet (ND). 8 weeks after HFD, animals were sacrificed, and both plasma CTRP9 and platelet aggregation were determined. HFD-fed animals increased weight gain significantly, and became hyperglycemic and hyperinsulinemic 8 weeks post-HFD. Compared to ND animals, HFD animals exhibited significantly decreased plasma CTRP9 concentration and increased platelet response to ADP, evidenced by augmented aggregation amplitude, steeper aggregation slope, larger area under the curve, and shorter lag time (P < 0.01). A significant negative correlation between plasma CTRP9 concentration and platelet aggregation amplitude was observed. More importantly, in vitro pre-treatment with CTRP9 significantly inhibited ADP-stimulated platelet activation in platelet samples from both ND and HFD animals. Taken together, our results suggest reduced plasma CTRP9 concentration during diabetes plays a causative role in platelet hyper-activity, contributing to platelet-induced cardiovascular damage during this pathologic condition. Enhancing CTRP9 production and/or exogenous supplementation of CTRP9 may protect against diabetic cardiovascular injury via inhibition of abnormal platelet activity.

## Background

Anti-platelet treatment, such as acetylsalicylic acid and aspirin, reduces cardiovascular morbidity and mortality [1]. It is well known type 2 diabetic patients are at very high cardiovascular risk [2], and should thereby potentially benefit significantly from anti-platelet treatment. Unfortunately, recent large clinical trials have demonstrated acetylsalicylic acid therapy less effectively prevents cardiovascular events in diabetic patients compared to normoglycemic individuals [3]. Moreover, concomitant type 2 diabetes increases the risk of high on-aspirin platelet reactivity (HPR), defined as inadequate inhibition of platelet function [4]. The pathologic mechanisms leading to enhanced platelet activity and reduced platelet response to therapeutic interventions in type 2 diabetes are complex and remain incompletely understood.

An adipokine secreted by adipose tissue [5, 6], adiponectin contains a stalk with 22 collagen repeats and a highly conserved globular domain. Typically present in plasma at concentrations up to 30 µg/ml, adiponectin is markedly down-regulated in association with obesity-linked diseases such as coronary artery disease and type 2 diabetes [7]. Clinical observations have revealed that plasma total adiponectin concentrations are inversely correlated with myocardial infarction risk [8, 9]. Moreover, adiponectin has been reported to have potent anti-platelet action, reducing vascular injury caused by abnormal thrombolysis [10–12]. Adiponectin knockout mice have been generated and studied by many groups. When metabolically challenged (e.g., high-fat diet), adiponectin-null mice develop insulin resistance, endothelial dysfunction, and vascular injury [13]. However, in the absence of dietary or metabolic stress, these animals show a relatively modest phenotype, suggesting potent compensatory mechanisms are in place.

Recently, a highly conserved family of adiponectin paralogs has been discovered, and designated the C1q tumor necrosis factor (TNF) related proteins (CTRPs). Exhibiting a similar structure as APN, each CTRP consists of four distinct domains including a N-terminal signal peptide, a short variable domain, a collagen-like domain, and a C-terminal C1q-like globular domain [14, 15]. Of all CTRPs identified to date, CTRP9 shares the greatest amino acid overlap with APN in its globular C1q domain [16]. We recently demonstrated that CTRP9 is an endothelium-dependent vasodilator and CTRP9 treatment attenuates diabetes-induced endothelial dysfunction [17]. Moreover, others and we have recently demonstrated CTRP9 protects the heart against acute ischemic/reperfusion injury [18, 19]. Most importantly, a recent study demonstrated that, distinctly different from adiponectin-knockout (which lacks phenotypic change under physiologic conditions), CTRP9-knockout mice gain more weight with normal diet and develop spontaneous insulin resistance and type 2 diabetes [20]. In contrast, CTRP9 transgenic mice are protected from diet-induced obesity and metabolic dysfunction [21]. These results suggest CTRP9 likely possesses more important metabolic regulatory function than other adipokines. However, the role of CTRP9 in dysregulated platelet hemostasis under diabetic conditions has not been previously investigated.

Therefore, the aims of this study were: (1) to determine the relationship between plasma CTRP9 alteration and platelet aggregation in high-fat diet induced diabetic animals, and (2) to investigate the effect of CTRP9 upon platelet aggregation.

## Methods

### High-fat diet induced diabetes

We utilized a previously established high-fat diet induced type 2 diabetes model to mimic Western diet-induced obesity/diabetes in human [22]. In brief, adult (8-week old) male C57BL/6 J mice were randomized to receive high-fat diet (HFD, 60 % kcal, Research Diets Inc. D12492i) or normal diet (ND, 10 % kcal control, D12450Bi) containing the same protein content as HFD. All experiments in this study were performed in adherence with the National Institutes of Health Guidelines on the Use of Laboratory Animals, and were approved by the Fourth Military Medical University Committee on Animal Care.

### Metabolic characterization

Mice were fasted overnight by removal to a clean cage without food at the end of their dark (feeding) cycle, approximately 6 p.m.. Mice were weighed at 8 a.m. the next morning. 30 µl blood was obtained via tail clip to assess plasma glucose (Accu-Chek Active Blood Glucose Monitoring System, Roche Diagnostics, Indianapolis, IN), plasma insulin (ELISA, Linco, Billerica, MA) and plasma CTRP9 (ELISA, Aviscera Bioscience, Santa Clara, CA). Weight and plasma measurements were recorded initially and every other week thereafter. The Homeostatic Model Assessment-Insulin Resistance (HOMA-IR), a surrogate measure of insulin resistance, was calculated initially and weekly thereafter, via HOMA calculator v2.3 (University of Oxford).

### Platelet preparation

Blood was obtained by intracardiac puncture from HFD or ND mice. Blood was drawn into polypropylene syringes containing one-tenth volume of 0.11 M sodium citrate, and centrifuged at 80 g for 10 min to obtain the platelet rich plasma (PRP), or 2400 g for 20 min to obtain platelet poor plasma (PPP).

### Platelet aggregation assay

500 µl of reference (PPP) or samples (PRP) was added to P/N 312 cuvettes (Chronolog Corp. Havertown, PA), and inserted into aggregometer wells (Chronolog Corp). 10 µM ADP reagent (Chronolog Corp) was added to the samples to induce platelet aggregation. To determine the effect of CTRP9 on platelet aggregation, recombinant murine CTRP9 (4 µg/ml, Aviscera Bioscience, Santa Clara, CA) was added in PRP for 20 min at 37 °C before ADP addition. CTRP9 is highly conserved throughout evolution. Mouse CTRP9 and its corresponding human ortholog share 100, 85, and 89 % amino acid identity in their short N-terminal variable regions, collagen domains, and C-terminal globular domains, respectively [16]. The amplitude, slope, area under the curve, and lag time of platelet aggregation was calculated automatically by manufacturer-provided software (Chronolog Corp).

## Statistical analysis

All values in the text and figures are presented as the mean ± SEM of n independent experiments. Data were analyzed by unpaired *t* test with GraphPad Prism 6 statistic software (La Jolla, CA). *P* values less than or equal to 0.05 (2-sided) were considered statistically significant.

## Results

### Plasma CTRP9 levels were significantly reduced in HFD induced type-*2 diabetic mice*

**Fig. 1:**
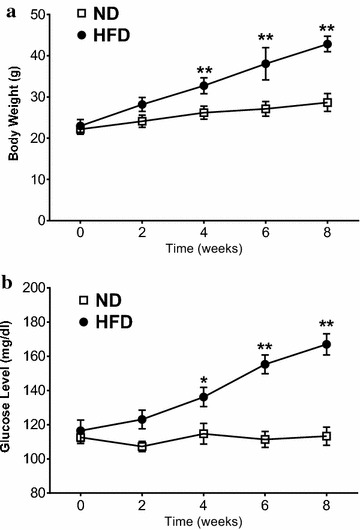
High fat diet (HFD) caused significantly greater weight gain (**a**) and hyperglycemia (**b**). N = 15 group. *P < 0.05, **P < 0.01 vs. normal diet (ND)

**Fig. 2:**
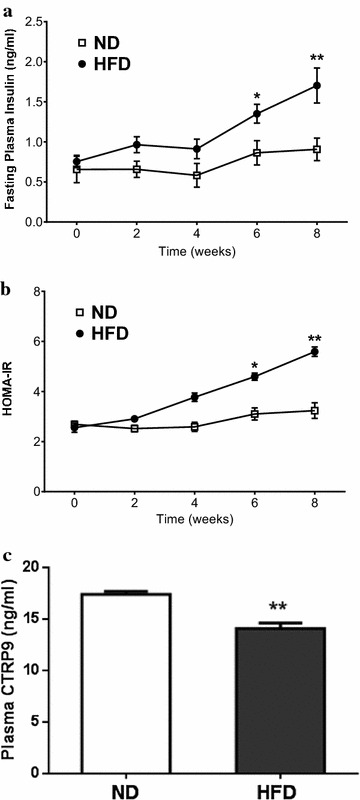
HFD significantly increased plasma insulin concentration (**a**) and HOMA-IR (**b**), and reduced plasma CTRP9 concentration (**c**). N = 15 group. *P < 0.05, **P < 0.01 vs. ND

To confirm establishment of type 2 diabetes by HFD, body weight, plasma glucose, and insulin concentration were determined. HFD mice had greater body weight increase compared to normal diet (ND) (Fig. 1). After 2 weeks, fasting plasma glucose increased steadily in HFD mice. 8 weeks post-HFD, fasting plasma insulin was significantly increased (Fig. 2a) and plasma CTRP9 concentration was significantly reduced (Fig. 2b).

### Platelet aggregation is significantly increased in diabetic animals and negatively correlated with plasma CTRP9

**Fig. 3:**
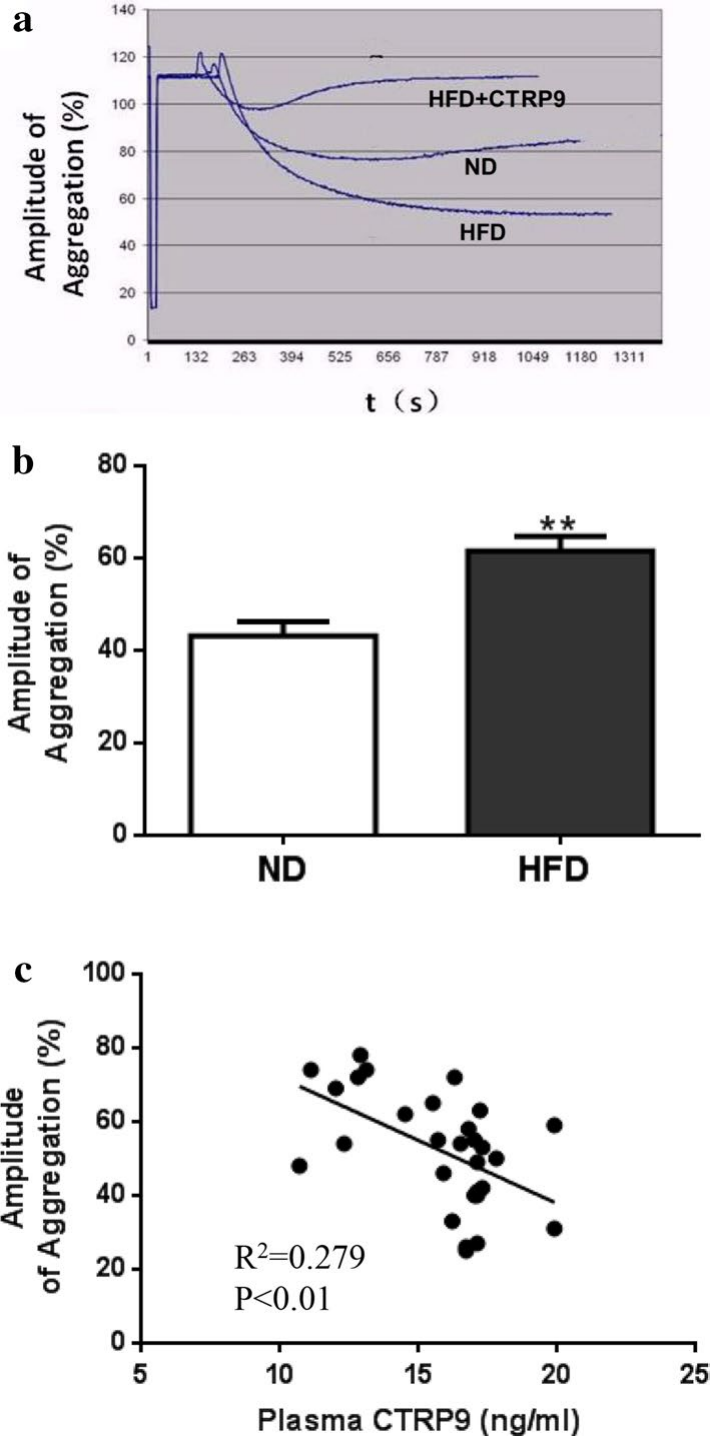
ADP-stimulated platelet aggregation is significantly increased in HFD animals. **a** Typical platelet aggregation tracing. **b** *Bar graph* summarizing data from 15 animals/group. **c** Significant negative correlation between plasma CTRP9 concentration and platelet aggregation was observed. *P < 0.05, **P < 0.01 vs. ND

**Fig. 4:**
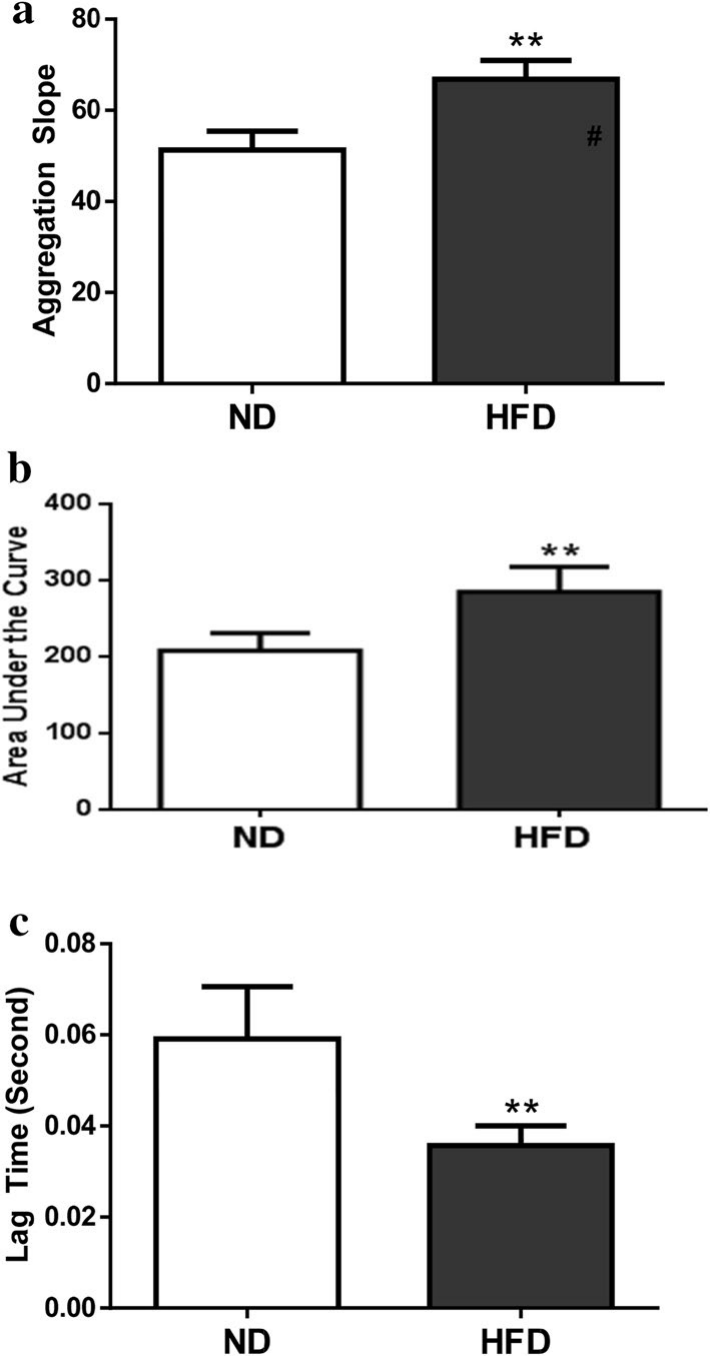
Both the platelet aggregation slope (**a)** and area under the curve (**b)** were significantly increased, but the lag time (**c)** was significantly reduced in platelet samples isolated from HFD animals. **P < 0.01 vs. ND

No significant difference was observed in total platelet counts between the two groups (987 ± 36 × 10^3^/µl vs. 966 ± 41 × 10^3^/µl). As illustrated in Fig. 3a and summarized in Fig. 3b, ADP-stimulated platelet aggregation was significantly increased in HFD mice samples. Importantly, a statistically significant negative correlation (P < 0.01) between plasma CTRP9 concentration and platelet aggregation was observed (Fig. 3c). To gain more insight into platelet activity alteration during diabetes, platelet aggregation slope, area under the curve, and lag time (reaction time after ADP addition) were determined. In HFD platelet samples, platelet aggregation slope and area under the curve were significantly increased, whereas the lag time was significantly reduced, compared to ND (Fig. 4), suggesting reduced plasma CTRP9 levels may contribute to abnormal platelet activity in diabetic animals.

### Recombinant CTRP9 significantly inhibited diabetic platelet aggregation

**Fig. 5:**
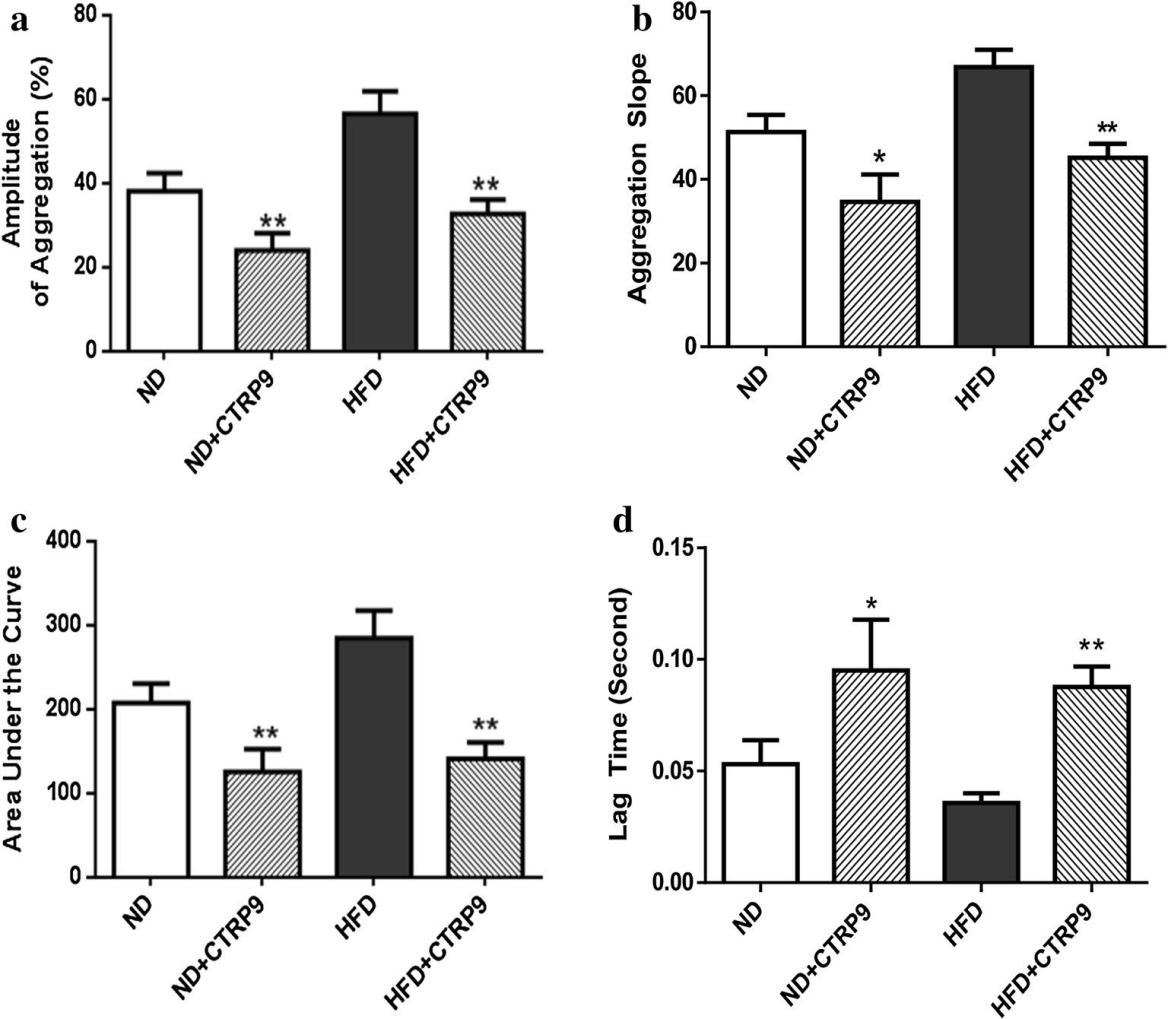
In vitro CTRP9 pre-treatment significantly reduced platelet function as evidenced by reduced platelet aggregation amplitude (**a**), decreased platelet aggregation slope (**b**), smaller area under the curve (**c**), and increased lag time in response to ADP (**d**). *P < 0.05, **P < 0.01 vs. respective control (without CTRP9 treatment)

To gain more direct evidence that CTRP9 may inhibit platelet function, the effect of in vitro CTRP9 pre-treatment upon ADP-induced platelet aggregation was determined. CTRP9 pre-treatment significantly reduced platelet function as evidenced by reduced platelet aggregation amplitude, decreased platelet aggregation slope, smaller area under the curve, and increased lag time in response to ADP (Fig. 5).

## Discussion

The current study made several significant observations. First, we demonstrated that plasma concentration of CTRP9, a novel cardioprotective adipokine, is significantly reduced in HFD-induced obesity/diabetic animals. This finding is consistent from a recent study reported by Peterson et al. [21]. Second, we provided the first evidence that platelet aggregation is negatively associated with plasma CTRP9 concentration. Third, we demonstrated for the first time that CTRP9 is a potent anti-platelet adipokine.

The role of platelets in thrombus formation is well known. Previous studies have reported an association between platelet activation and the degree of insulin resistance reflected by HOMA-IR [23]. Platelet hyper-reactivity is one of the most important causes of accelerated atherosclerosis and increased thrombotic risk in diabetic patients [24], contributing to a 2- to 4-fold increased coronary artery disease risk. During platelet activation, arachidonic acid is released from membrane phospholipids, which is oxygenated into thromboxane A2, a potent pro-aggregatory and vaso-constricting compound. Moreover, a previous study has shown that increased platelet aggregation is already detectable in diabetic patients without apparent vascular complications [25], indicating abnormal platelet reactivity may play a causative role in diabetic vascular morbidity and mortality. Therefore, identifying the molecular mechanisms responsible for abnormal platelet activity during diabetes may have great translational benefit.

Adiponectin is the most extensively investigated cardioprotective adipokine [26–29]. Hypoadiponectinemia correlates with increased AMI risk [30–32], and poorer cardiac functional recovery after MI with reperfusion [33, 34]. Exogenous APN supplementation significantly protects the heart against ischemic injury [35, 36]. However, the cardioprotective effects of APN are significantly attenuated in diabetic animals [22]. Moreover, complete APN abrogation results in only mild phenotypic change, unless pathologically challenged (e.g., by high-fat diet or ischemia), suggesting existent overlapping regulators. Efforts to identify such regulators have led to the discovery of a family of APN paralogs, designated the C1q/TNF-related proteins (CTRP1–CTRP15) [14, 37–40]. Although the CTRP family member roster has rapidly grown since their initial discovery 9 years ago, the biological functions of CTRPs have only been realized in recent years [41–44]. Thus far, most published studies focus upon the beneficial metabolic-regulatory functions of CTRPs [15, 16, 45]. Among these studies, CTRP12 is an insulin-sensitizing, anti-inflammatory adipokine, downregulated by obesity [46].

In the current study, we demonstrated plasma CTRP9 concentration is negatively correlated with ADP-induced platelet aggregation. Moreover, we demonstrated that in vitro CTRP9 pre-treatment significantly inhibited platelet activity. It should be indicated that in vitro CTRP9 concentration utilized in this study to inhibit ADP-induced platelet aggregation is much higher than in vivo physiological concentration. This concentration was selected because in vitro platelet aggregation was induced by high concentration of ADP which causes much stronger platelet aggregation than that seen in vivo. As such, higher concentration of CTRP9 is required to achieve a significant inhibition. More importantly, CTRP9 is the closest paralog of adiponectin which circulates in plasma at concentration greater than 10 µg/ml. As adiponectin concentration is significantly reduced in diabetic individuals but biological response to exogenous adiponectin is significantly impaired, supplementation of CTRP9 at super-pharmacological concentration, such as that used in our in vitro platelet inhibition experiment, may represent an effective therapeutic intervention against diabetic pathology.

Taken together, our results suggest that reduced plasma CTRP9 concentration during diabetes plays a causative role in platelet hyper-activity, contributing to platelet-induced cardiovascular damage. Enhancing CTRP9 production and/or exogenous supplementation of CTRP9 may protect against diabetic cardiovascular injury via inhibition of abnormal platelet activity.

## Conclusion

Our results show that diabetes induces reduced plasma CTRP9 concentration, which plays a causative role in platelet hyper-activity and subsequent platelet-induced cardiovascular damage during this pathologic condition. Supplementation of exogenous CTRP9 may provide a protection against diabetes induced cardiovascular injury via inhibition of platelet hyper-reactivity.
